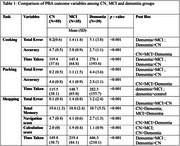# Performance Based Assessment: A unique approach to ecologically valid assessment of cognition

**DOI:** 10.1002/alz70857_107157

**Published:** 2025-12-25

**Authors:** Keerthana Bengaluru Somashekar, Aparna Lalana Venugopal, Nithin Thanissery, Susmita Patra, Riya Rafeekh, Faheem Arshad, Atanu Biswas, Suvarna Alladi

**Affiliations:** ^1^ National Institute of Mental Health and Neurosciences, Bengaluru, Karnataka, India; ^2^ Bangur Institute of Neurosciences and IPGME&R, Kolkata, West Bengal, India; ^3^ School of Liberal Arts and Sciences, RV University, Bengaluru, Karnataka, India

## Abstract

**Background:**

Impairments in daily activities often signal deficits in cognitive abilities. Existing tests evaluating cognition rely heavily on paper‐pencil formats that favor educated populations. Ecologically valid, person‐centered tests based on observing natural actions are needed to address these limitations. This study aims to develop and validate Performance Based Assessment (PBA) to identify and understand deficits in real‐life situations for MCI and dementia patients.

**Method:**

A scoping review was conducted and suitable PBA was developed to assess executive functions in individuals with cognitive impairment. Three tasks: cooking task, packing a bag task and shopping tasks were finalised based on feasibility and applicability of tasks. Expert validation was conducted and necessary changes to the tasks were made. Tasks were scored on total errors made, accuracy rating based on total errors and total time taken to complete the task. Total errors were a sum of errors such as comprehension, omission, commission, estimation and other observed errors. Cognitively normal (CN) individuals, MCI and dementia patients recruited from Cognitive Disorders Clinic (CDC), NIMHANS, matched for age and gender, completed sociodemographic assessments, ACE‐III, CDR, and IADL evaluations, along with familiarity and ease ratings of each task.

**Result:**

Interrater reliability of scoring and error coding was established by 2 independent examiners and Internal consistency using Cronbach's alpha was >0.7 for all 3 tasks. In cooking and packing task, dementia group had highest total errors and time taken with lowest accuracy compared to CN and MCI. Omission error was most observed type of error in both tasks. Dementia group had reduced ability to carry out the cooking task compared to MCI and controls (Table 1). Outcome measures negatively correlated to total ACE‐III score, Global CDR score and Cognitive Disability Index. No significant effect of gender, occupational status, languages known, literacy and community dwelling were observed on outcome variables in controls, MCI and dementia groups.

**Conclusion:**

The PBA successfully differentiated between CN, MCI, and dementia groups. Tasks proved relevant across gender, literacy, and socioeconomic backgrounds, supporting their use in diverse societies like India. Expanding into clinically relevant, naturalistic tasks will enhance detection of cognitive impairment in complex daily activities.